# Valorisation of the Halophyte *Cakile maritima* as a Food Resource for Human Consumption

**DOI:** 10.3390/foods15183261

**Published:** 2026-09-15

**Authors:** Ricardo Mir, María Dolores García-Martínez, Monica Boscaiu, Oscar Vicente, Jaime Prohens, María Dolores Raigón-Jiménez

**Affiliations:** 1 Institute for the Conservation and Improvement of Valencian Agrodiversity (COMAV), Universitat Politècnica de València, Camino de Vera s/n, 46022 Valencia, Spain; magarma8@qim.upv.es (M.D.G.-M.); ovicente@upvnet.upv.es (O.V.); jprohens@btc.upv.es (J.P.); mdraigon@qim.upv.es (M.D.R.-J.); 2 Mediterranean Agroforestry Institute (IAM), Universitat Politècnica de València, Camino de Vera s/n, 46022 Valencia, Spain; mobosnea@eaf.upv.es

**Keywords:** *Cakile maritima*, halophytes, sensory tasting, saline agriculture, nutritional profile

## Abstract

Increasing soil salinisation challenges food production, since most crops are highly sensitive to salinity. The identification of wild halophytes adapted to saline environments with nutritional value represents a promising strategy for food production on salt-affected farmlands. Assessing the nutritional potential of such species requires evaluating their proximate composition, mineral profile, and antioxidant properties. In parallel, citizen-science approaches complement and enhance scientific research by actively engaging potential final consumers in the research process, thereby improving the societal relevance, dissemination, and potential impact of scientific findings. In this study, we characterised the nutritional profile of the facultative halophyte *Cakile maritima* and found it to be comparable, and in some respects superior, to that of other conventional leafy vegetables, particularly regarding its mineral composition and bioactive compounds. Moreover, similar though not identical nutritional characteristics were observed in two *C. maritima* leaf morphotypes analysed, since differences in dry matter, ashes, total proteins and carbohydrates were identified. Interestingly, vitamin C accumulation was organ- and morphotype-dependent. Finally, the biochemical characterisation was complemented with an online survey that showed a clear predisposition of consumers towards the incorporation of wild edible plants into their diet, together with a sensory evaluation in which 32 participants assessed the acceptability of up to 11 dishes prepared using *C. maritima*. Sensory evaluation revealed a prominent bitter flavour amongst dishes containing *C. maritima*, with weighted scores for negative perceptions slightly exceeding those for positive ones, suggesting that its sensory profile may limit its acceptance by the general public while offering potential for specific consumer segments. Overall, our findings highlight the nutritional potential of *C. maritima* and support its valorisation as a sustainable species for saline agriculture.

## 1. Introduction

Global agriculture is currently facing unprecedented challenges derived from climate change, land degradation, and the progressive salinisation of soils and water resources. Rising temperatures, altered precipitation patterns, and increasing seawater intrusion are accelerating natural or primary salinisation of soils, particularly in coastal areas and arid and semi-arid regions [1,2]. This problem is particularly pronounced in irrigated farmlands due to anthropogenic or secondary salinisation caused by inappropriate fertiliser application and the use of brackish irrigation water. Indeed, more than 20% of the world’s irrigated agricultural land is already affected by salinity, with a substantial increase expected in the coming decades [2]. Under these conditions, conventional glycophytic crops exhibit reduced productivity or complete crop failure, threatening food security and agricultural sustainability. On the other hand, the increasing homogenisation of the global food supply, characterised by a convergence of national diets toward a limited number of dominant crops, particularly major cereals and oil crops, represents a structural risk to food security under climate change [3].

In this context, the diversification of food systems through the incorporation of resilient wild plant species represents a promising and, in some cases, unavoidable strategy. Halophytes, defined as plants capable of completing their life cycle in environments with high salt concentrations, are able to activate disparate physiological and biochemical mechanisms to deal with salinity more effectively and rapidly than salt-sensitive glycophytes. Halophytes efficiently detoxify the reactive oxygen species (ROS) generated under salinity through a complex antioxidant system involving both enzymatic activities and non-enzymatic compounds, including ascorbate, glutathione, phenolic compounds, carotenoids and tocopherols. Among these non-enzymatic antioxidants, polyphenols are a diverse group of plant secondary metabolites that interact with ROS and alleviate oxidative stress [4]. Ascorbic acid (vitamin C) is another component of non-enzymatic plant redox metabolism, occurring in all major plant organs, with particularly high concentrations in photosynthetically active tissues, meristems and developing fruits, where it functions as a major antioxidant and signalling molecule regulating growth, development, photosynthesis and responses to biotic and abiotic stress [5]. Consequently, plant species with high concentrations of antioxidant compounds may be considered valuable from a nutritional perspective, as their consumption has been associated with beneficial effects on human health by reducing oxidative stress and preventing chronic diseases [6].

In addition to their ability to grow and develop under salt stress, many halophytes exhibit favourable nutritional profiles, containing valuable minerals, dietary fibre, proteins, fatty acids and bioactive compounds with antioxidant properties, and have attracted increasing attention as alternative or complementary crops for saline agriculture [7,8]. Consequently, halophytes are increasingly recognised not only as tools for land restoration and haloremediation but also as potential food resources for human consumption [9,10]. Amongst edible halophytes, *Cakile maritima* Scop. (Brassicaceae), commonly known as sea rocket, is an annual species widely distributed along coastal dunes and sandy beaches of Europe, North Africa, Australia and North America [11]. This species is well adapted to nutrient-poor and unstable substrates, exhibiting tolerance to salt spray, soil salinity and drought [12,13,14]. Traditionally, *C. maritima* has been consumed sporadically in some Mediterranean regions, mainly as a wild leafy vegetable, raw or lightly cooked, and occasionally used as a condiment due to its pungent flavour typical of Brassicaceae [15,16]. However, despite its wide distribution and resilience, *C. maritima* remains largely underutilised and poorly characterised as a food crop, and in the past it was mostly used during famine periods [16]. Furthermore, given the invasive behaviour of *C. maritima* in some regions, its valorisation as a food resource could offer an additional management opportunity, as harvesting wild populations for human consumption may contribute to limiting their expansion [17].

Recent studies have begun to highlight the nutritional and functional potential of *C. maritima*. Leaves and aerial tissues have been reported to contain appreciable amounts of proteins, dietary fibre and essential minerals, as well as phenolic compounds and vitamin C, which contribute to antioxidant capacity [18,19]. Furthermore, the species exhibits remarkable plasticity in its biochemical composition in response to environmental conditions, particularly salinity, which may enhance the accumulation of health-promoting metabolites [20,21,22]. Seed oils of *C. maritima* have also been investigated for their fatty acid composition, suggesting possible applications beyond fresh consumption [15,23]. Nevertheless, comprehensive evaluations encompassing multiple plant organs, nutritional fractions and morphotypes remain scarce.

Despite growing scientific interest, the successful incorporation of wild halophytes into human diets depends not only on their agronomic performance and nutritional properties, but also on social acceptance, culinary versatility, and consumer perception. In this regard, citizen-science approaches and participatory research are increasingly recognised as valuable tools to bridge the gap between laboratory research and real-world food systems [24]. Surveys assessing attitudes towards wild edible plants, as well as sensory evaluation and tasting sessions, will provide essential information on consumer willingness to adopt novel foods and identify potential barriers to their acceptance. On the other hand, sensory evaluation plays a pivotal role in food science, as consumer acceptance of novel foods depends not only on their nutritional and functional properties but also on their sensory characteristics, including appearance, aroma, flavour, and texture. Indeed, several recent studies incorporate such approaches to assess the acceptance of wild plant species as alternative food resources [25,26], including halophytes [27]. These tasting sessions use data from untargeted volunteers as participants [26,28], or from culinary professionals or trained tasters [25,29,30].

The present study aims to contribute to the valorisation of *C. maritima* as a food resource for human consumption by combining detailed laboratory biochemical analyses with a citizen-science approach. Specifically, we performed a comprehensive proximate analysis of different plant organs (i.e., stems, leaves, fruits, roots and flowers), including quantification of dry matter, moisture, fibre, ash content, and the main biomolecules (carbohydrates, proteins and lipids). Moreover, we characterised the mineral composition of these organs, focusing on macro- and micronutrients relevant to human nutrition, including sodium, potassium, calcium, magnesium, phosphorus and iron, amongst others. Furthermore, the antioxidant potential of *C. maritima* was assessed by measuring total antioxidant capacity, vitamin C accumulation, and total polyphenol content in selected edible organs. Importantly, all these parameters were separately measured in the two main leaf morphotypes of *C. maritima*, characterised by pinnatifid or entire leaves, to explore potential intraspecific variability of nutritional interest. To complement the biochemical analyses, an online survey was conducted to evaluate public perception and acceptance of wild plants as food resources. Finally, a controlled tasting session involving 32 participants was conducted, during which up to eleven different food preparations based on *C. maritima*, including starters, main dishes, desserts and teas, were evaluated.

By integrating analytical chemistry, plant physiology and citizen science, this work seeks to provide a holistic assessment of *C. maritima* as an underexploited halophytic food species. Our results aim to support the inclusion of this species in diversified and resilient food systems adapted to saline environments, highlighting its role not as a substitute for existing crops but as a complementary component within saline and transitional agricultural landscapes.

## 2. Materials and Methods

### 2.1. Plant Material and Sampling

**Figure 1:**
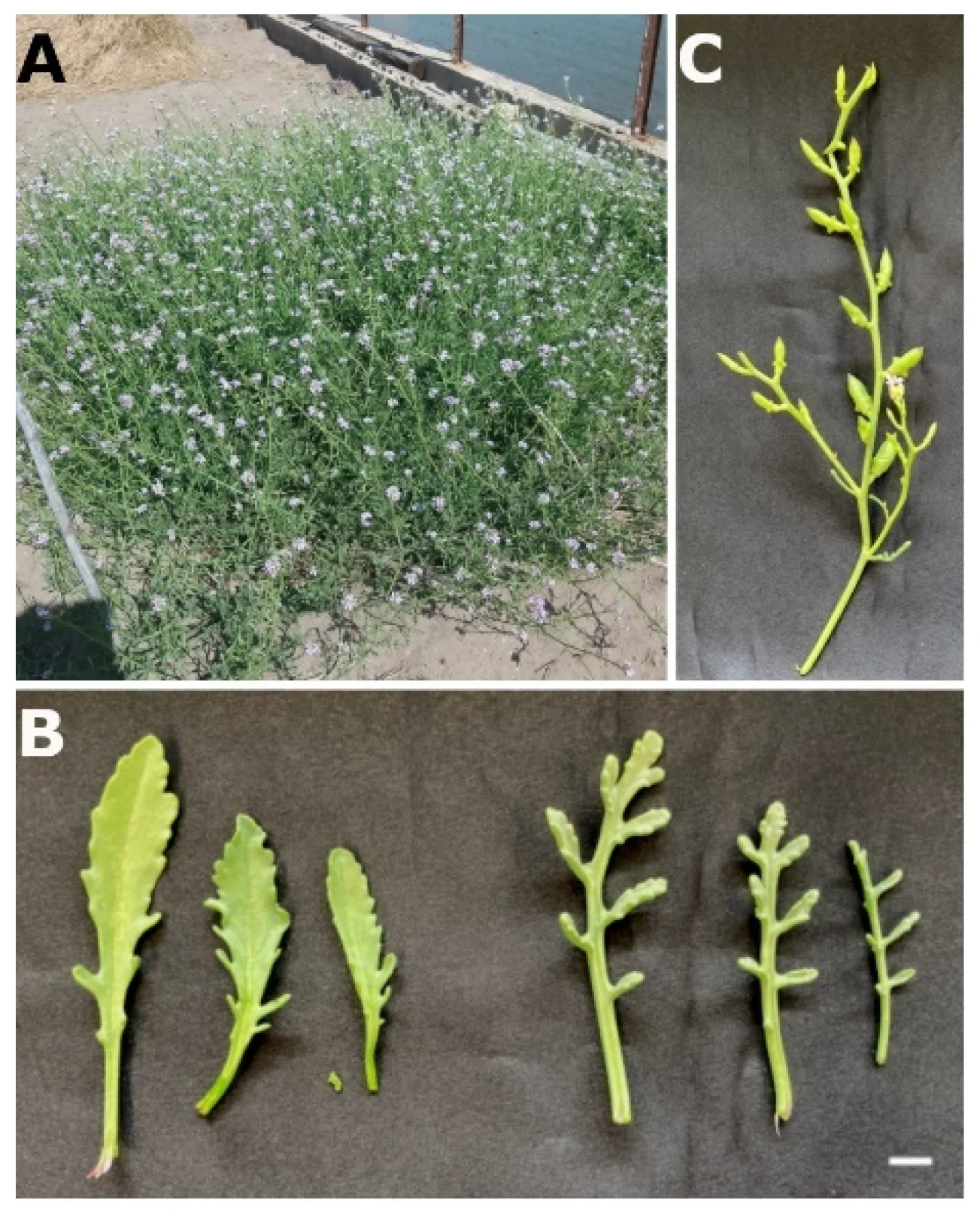
(**A**) *Cakile maritima* populations grown on sandy plots near water ponds. (**B**) Representative images of leaves of entire lamina morphotype (left) and pinnatifid morphotype (right) sampled for nutritional analysis. (**C**) Representative image of immature fruits sampled for nutritional analysis.

Seeds of *Cakile maritima*, collected along the Mediterranean coast of Spain (Platja del Serradal, Castelló de la Plana), were supplied by the Botanical Garden of Valencia, Spain. To reproduce natural populations in dune ecosystems, *C. maritima* seeds were randomly sown on sandy plots located near the Albufera lake, specifically in El Palmar, Valencia (39°18′35″ N 0°19′06″ O), forming small plant populations situated near water ponds (Figure 1A). Plants were naturally grown with no artificial water supply until flowering and the initiation of fruit formation. The plant population included both entire lamina and pinnatifid leaf morphotypes (Figure 1B). Sampling was conducted on 27 May 2024. Average temperature along this month (from the 1 May to harvesting time) was registered as 19.7 ± 0.3 °C, with average maximum and minimum temperatures of 26.1 ± 0.5 °C and 12.9 ± 0.5 °C, respectively, and average relative humidity during this period was registered as 60.1 ± 2.5%, according to Agencia Estatal de Meteorología (AEMET; https://servicio.mapa.gob.es/websiar/ResultadoConsultaDatos.aspx, accessed on 16 October 2024).

Plant material, including leaves (Figure 1B), stems, and immature fruits (Figure 1C), was harvested separately from plants with entire lamina leaves (*n* = 3) or pinnatifid leaves (*n* = 3). Accordingly, when leaf morphotypes are not distinguished, results are based on six samples (*n* = 6), whereas when leaf morphotypes are reported separately, each group comprises three samples (*n* = 3). No distinction between leaf morphotypes was made when harvesting roots and flowers (*n* = 3). A total of three biological replicates were used per measurement. Each fresh vegetal sample was divided into three fractions. One was used to obtain the dry sample, which was subsequently ground (Retsch GmbH, Haan, Remscheid, Germany) until a homogeneous mixture was obtained, with an approximate particle size of 250 µm. The resulting powder of the dried and ground plant material was stored in high-density polyethylene bottles at room temperature and darkness until further analysis. This fraction was used to determine the proximate and mineral composition. A second fraction of the fresh plant material was used to obtain a methanolic extract to determine the total antioxidant capacity, and the third fraction was used to prepare an aqueous extract to measure total polyphenol and vitamin C contents.

### 2.2. Proximate Composition Analysis

Proximate composition in the plant materials was determined by following the recommendations of the Association of Official Analytical Chemists [31]. Moisture and dry matter was determined by gravimetric analysis using a stove J.P. Selecta (Barcelona, Spain) at 70 ± 0.1 °C until constant weight (AOAC 984.25 method); nitrogen by the Kjeldahl method (AOAC 984.13 method) using the Foss Tecator 2100 Kjeltec distillation unit (Hillerød, Denmark), and to calculate protein from nitrogen, the factor 6.25 was used [32]; fat using a Foss Soxhlet model ST243 SoxtecTM from LabtecTM line (Hillerød, Denmark; AOAC 983.23 method); fibre quantifying the residue that persists after two successive hydrolyses, one acidic and the other alkaline (AOAC 991.43 method); ashes by calcination in a Carbolite CWF 1100 chamber furnace at 550 °C (AOAC 923.03 method). The total carbohydrate amount was obtained by subtracting the amounts of water, protein, total fat, fibre and ash from 100. The analyses were done in triplicate. Results are expressed as percentages (g·100 g^−1^) of fresh weight (fw) or dry weight (dw), as indicated.

### 2.3. Mineral Composition Analysis

The individual mineral composition of the plant materials was determined by inductively coupled plasma emission spectroscopy (ICP-OES) from ashes dissolved and settled with concentrated HCl until a final concentration of 2% HCl. The equipment used was an Agilent 710 ICP-OES (Agilent Technologies, Mulgrave, Victoria, Australia). The wavelengths selected for each element were the following: 184.887 for Hg, 196.026 nm for Se, 213.857 nm for Zn, 228.802 nm for Cd, 238.204 nm for Fe, 249.678 nm for B, 257.610 nm for Mn, 281.615 nm for Mo, 285.213 nm for Mg, 317.933 nm for Ca, 324.754 nm for Cu, 405.781 nm for Pb, and 769.897 nm for K. A simple flame photometer (Jenway PF7, Jenway, Essex, UK) with filter for sodium was used for the determination of this element in plant materials (AOAC 985.35 method). Phosphorus was determined using a spectrophotometric method based on the formation of a yellow phosphomolybdovanadate complex in an acidic medium in the presence of V^5+^ and Mo^6+^ ions. Absorbance was measured at 430 nm, following the procedure described by [31]. In all cases, the analyses were done in triplicate. Results are expressed in mg of the mineral element per 100 g of dw or fw, as indicated.

### 2.4. Vitamin C, Total Antioxidant Capacity and Total Polyphenols Quantification

Vitamin C was determined reflectometrically, based on the reduction of yellow molybdophosphoric acid to phosphomolybdene blue. The equipment used was a reflectometer RQflex plus and a Reflectoquant test for ascorbic acid (Merck, Darmstadt, Germany). Samples of 15 g of fresh plant material were homogenised in an industrial blender by adding distilled water to facilitate blending. After homogenisation, the liquid was filtered with a mesh (0.5 mm) into a 100 mL test tube, which was filled with distilled water up to 50 mL. The results were expressed in mg of ascorbic acid per 100 g fw.

Total antioxidant capacity (TAC) determination in plant materials was performed according to previously described methodology [33] based on the capture of the free radical 2,2-diphenyl-1-picrylhydrazyl (DPPH). A methanolic extract was obtained by mixing 0.8 g of plant material in 5 mL methanol solution (80% *v*/*v*); it was stirred for 1 h at room temperature, using an orbital shaker (Stuart Scientific, Chelmsford, Essex, UK). A volume of 3.9 mL of the DPPH solution (25 ppm in methanol) was mixed with 0.1 mL of the methanolic extract, and the absorbance was measured at 515 nm after 45 min of incubation with the DPPH solution in the dark, with a spectrophotometer (Schott UV line 9400, Essex, UK). The antioxidant Trolox was used as standard, and the results were expressed as micromoles of Trolox equivalents per 100 g of fresh weight (µmol TE·100 g^−1^ fw).

Total polyphenols were determined by reaction with the Folin–Ciocalteu reagent, according to [34]. An aliquot (50 µL) of aqueous extract obtained by grinding the plant materials with distilled water in a ratio of 2:1 (solvent:plant) was mixed with 500 µL of the reagent (previously diluted with water 1:10 *v*/*v*). Upon 5 min incubation, 500 µL of 6% (*w*/*v*) Na_2_CO_3_ solution was added to each sample. The mixture was agitated for 10 s and allowed to stand for 1 h at room temperature in the dark for colour development. The absorbance at 750 nm was measured by a spectrophotometer (Jenway 6715/UV-V, Stone, UK), using gallic acid as a standard. The results were expressed as mg gallic acid equivalents per 100 g of fresh weight (mg GAE 100 g^−1^ fw).

Antioxidant capability and compound concentrations were expressed on a fresh-weight basis (FW) to reflect their content in the fresh plant material intended for human consumption.

### 2.5. Online Survey

An online survey, comprising both closed- and open-ended questions, was developed to collect data on consumers’ predisposition to incorporate resilient wild plant species such as *C. maritima* in their diet. For this purpose, an open platform (i.e., Google Forms) was used. The questionnaire was distributed via a digital messaging platform (i.e., WhatsApp) using a snowball sampling approach, whereby participants were encouraged to forward the survey link to other potential respondents. Participation was voluntary, and respondents’ anonymity and confidentiality were maintained throughout the study. Data collection was conducted between September and December 2025, and all responses were securely stored.

Data on participants’ dietary habits and perceptions regarding food production were collected through a structured questionnaire. Initially, participants were asked about their role in household grocery shopping and their familiarity with or interest in alternative or gourmet cuisines. Subsequently, they were asked to rate the importance of several aspects related to food products and plant-based food production using a five-point Likert scale, where 1 indicated the lowest level of importance and 5 the highest. The assessed aspects included key attributes influencing food choices as well as major concerns associated with plant-based food production. Participants were also asked to indicate the frequency with which they consumed different types of dishes using the same five-point scale.

To facilitate the visualisation of overall participant responses, the obtained raw data were converted into a weighted numerical score and represented graphically. For each response category, the assigned score (1–5) by participant was multiplied by the number of participants selecting that option. The overall score for each item was then calculated as the sum of these weighted values across all response categories.

### 2.6. Tasting Event of Dishes Elaborated with Cakile maritima

A meticulous menu of up to 11 dishes, including starters and salads, main and side dishes, and dessert and tea, was elaborated by the professional chef Paco Brocal Rubio. This menu included, in degustation order, (1) salmon tartare prepared with powdered dehydrated *C. maritima* leaves and garnished with fresh *C. maritima* flowers, (2) a caprese salad with raw *C. maritima* leaves, (3) salty omelette with *C. maritima* leaves and tender stems, (4) mayonnaise elaborated with Salicornia, as control *C. maritima*-free dish, (5) crystal bread prepared with powdered dehydrated *C. maritima* leaves with old beef garnished with *C. maritima* petals, (6) salty and (7) sweet pickle from *C. maritima* leaves and tender stems, (8) raw tender *C. maritima* leaves, (9) sweet omelette with *C. maritima* leaves and tender stems, (10) chocolate balls coated with powdered dehydrated *C. maritima* leaves, and (11) tea elaborated with tender *C. maritima* leaves.

A total of 32 participants took part in the sensory evaluation. Participants were recruited voluntarily through posters displayed at the Ciudad Politécnica de la Innovación (UPV) and word-of-mouth communication. From the applications received, participants were selected on a first-come, first-served basis. University students were excluded from the final sample. The participants were not formally trained in sensory analysis and therefore constituted an untrained consumer panel. The tasting event was conducted in two separate sessions held one week apart. The first session included 14 participants, and the second included 18 participants. In both sessions, all participants tasted the dishes simultaneously and in the same fixed order, from dish 1 to 11. All dishes were coded, and no information regarding their names or ingredients was provided to participants, except for potential allergen information.

To assess the acceptance of the prepared dishes, participants completed a sensory evaluation questionnaire comprising two sections. In the first section, participants were asked to indicate the dominant tastes detected (i.e., identified or perceived) in each dish (bitter, acidic, sweet, umami and salty), together with their intensity (light or intense). Moreover, participants were asked to indicate whether each flavour type within each bite was perceived as pleasant (positive) or undesirable (negative), and to rate the intensity of this perception (light or intense). Qualitative sensory responses were subsequently converted into quantitative scores for data analysis as follows. For taste intensity, a score of 1 was assigned to light perception and a score of 2 to intense perception. Similarly, for hedonic perception, scores of 1 and 2 were assigned to light and intense perceptions, respectively, either positive or negative. Scores were then summed across participants and the 11 dishes for each taste attribute and hedonic perception and compiled in tabular form.

In the second section, overall liking was evaluated using a linear hedonic scale, which allowed participants to indicate their degree of preference along a continuous oblique line. Responses were subsequently converted into numerical values ranging from 0 to 7 for graphical representation and statistical analysis, following the methodology described by [35].

### 2.7. Statistical Analysis

Statistical analyses were performed using GraphPad Prism version 8.1 (GraphPad Software, San Diego, CA, USA). Normal distribution of data was assessed by the Shapiro–Wilk test. Significant differences for normally distributed data were assessed using one-way analysis of variance (ANOVA). The Tukey post-hoc test (*p* < 0.05) was used to evaluate group differences after the null hypothesis was rejected. For non-normally distributed data, statistical differences were analysed by the Kruskal–Wallis test. To assess the significance of differences between morphotypes, pairwise *t*-tests (*p* <0.05) were used.

## 3. Results

### 3.1. Proximate Analysis of Different Cakile maritima Organs

**Figure 2:**
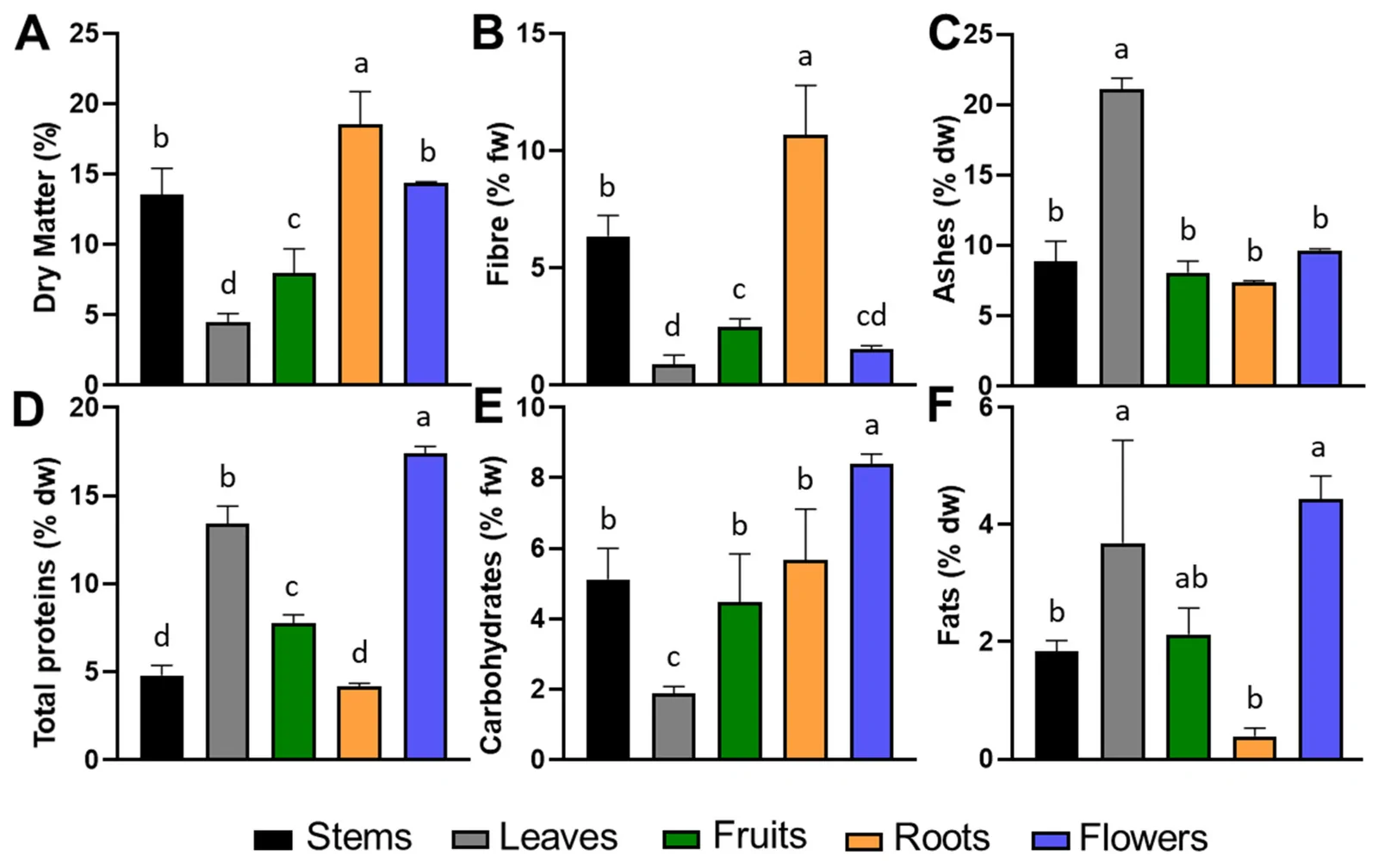
Proximate analysis of different edible organs of *Cakile maritima*. Bar charts show the values recorded for (**A**) dry matter, (**B**) fibre content, (**C**) ash content, (**D**) total protein, (**E**) total carbohydrates, and (**F**) fat, expressed as percentages, in stems, leaves, immature fruits, roots and flowers, as indicated. Values are average percentage values ± SE, referred to fresh weight (fw; fibre and carbohydrates) or dry weight (dw; ashes, total protein, and fats). For stems, leaves and fruit samples, *n* = 6; for root and flower samples, *n* = 3. Different letters above the bars indicate statistically significant differences according to one-way ANOVA followed by Tukey’s multiple comparison test (*p* < 0.05).

To study the overall nutritional properties of *Cakile maritima*, a proximate analysis approach on different plant organs, namely stems, leaves, fruits, roots and flowers, was conducted. First, dry matter content analysis showed statistical differences amongst organs. Roots were the driest organs, with a measured value of 18.6%, followed by stems (13.5%) and flowers (14.4%). Leaves and immature fruits presented the lowest dry matter content of all organs analysed, being 4.5% and 8%, respectively (Figure 2A). Next, total fibre was quantified to study the digestibility of different *C. maritima* organs. The results showed that fibre was notably lower in leaves (0.91% of fw), immature fruits (2.49% of fw) and flowers (1.54% of fw) than in stems (6.36% of fw) and roots (10.7% of fw; Figure 2B). Finally, measurements of total ash content revealed that the mineral fraction of leaves (21.1% of dw) was more than two-fold higher than those registered for stems (8.9% of dw), fruits (8.1% of dw), roots (7.4% of dw) and flowers (9.6% of dw; Figure 2C).

The mineral fraction was further analysed by quantifying micro- and macroelements in stems, leaves, fruits, flowers and roots, and was referred to either dry weight (Table 1) or fresh weight (Supplementary Table S1). Potassium was the most abundant mineral element in leaves, which also accumulated higher concentrations of sodium, calcium and magnesium than the other plant organs (Table 1). Importantly, from a nutritional perspective, 100 g of fresh *C. maritima* leaves would provide 220 mg of Ca (Supplementary Table S1), corresponding to approximately 28% of the UE Nutrient Reference Value (NRV) for calcium (800 mg per day [36]). On the other hand, sodium accumulation in leaves reached up to 680 mg/100 g of dw, about two-fold the values registered for stems, three-fold the values for roots, and four- and five-fold the values obtained for fruits and flowers, respectively. Conversely, phosphorus was highly accumulated in flowers (2118.37 mg/100 g dw) and leaves (1731.18 mg/100 g dw), significantly higher than in the other organs assayed (Table 1). These differences amongst organs in phosphorus accumulation were more noticeable in measurements relative to fresh weight and showed flowers to be especially enriched in this element (Supplementary Table S1). Several other microelements, such as iron, copper, zinc, boron and mercury, were more abundant in stems than in other organs, whereas manganese, molybdenum, and selenium resulted in non-statistically different concentrations amongst organs (Table 1). Cd concentration was under the limit of detection for all organs tested. Similar patterns for most mineral element profiles were observed when expressed as fresh weight (Supplementary Table S1).

The nutritional properties were further analysed by measuring the accumulation of the main biomolecules, total proteins, carbohydrates and fats, in different organs (Figure 2D–F). Protein accumulation registered in flowers was the highest amongst organs (17.4% of dw), followed by leaves (13.4% of dw), immature fruits (7.7% of dw) and finally, stems (4.8% of dw) and roots (4.2% of dw; Figure 2D). On the other hand, overall carbohydrate content was low compared to other crops, with those registered for flowers and leaves being the highest (8.4% of fw) and lowest (1.9% of fw), respectively (Figure 2E). Finally, fat concentration in roots presented the lowest value (0.4% of dw), compared to the rest of the organs analysed. The highest fat percentages were obtained for leaves (3.7% of dw) and flowers (4.4% of dw; Figure 2F).

### 3.2. Antioxidant Potential of Different Cakile maritima Organs

**Figure 3:**
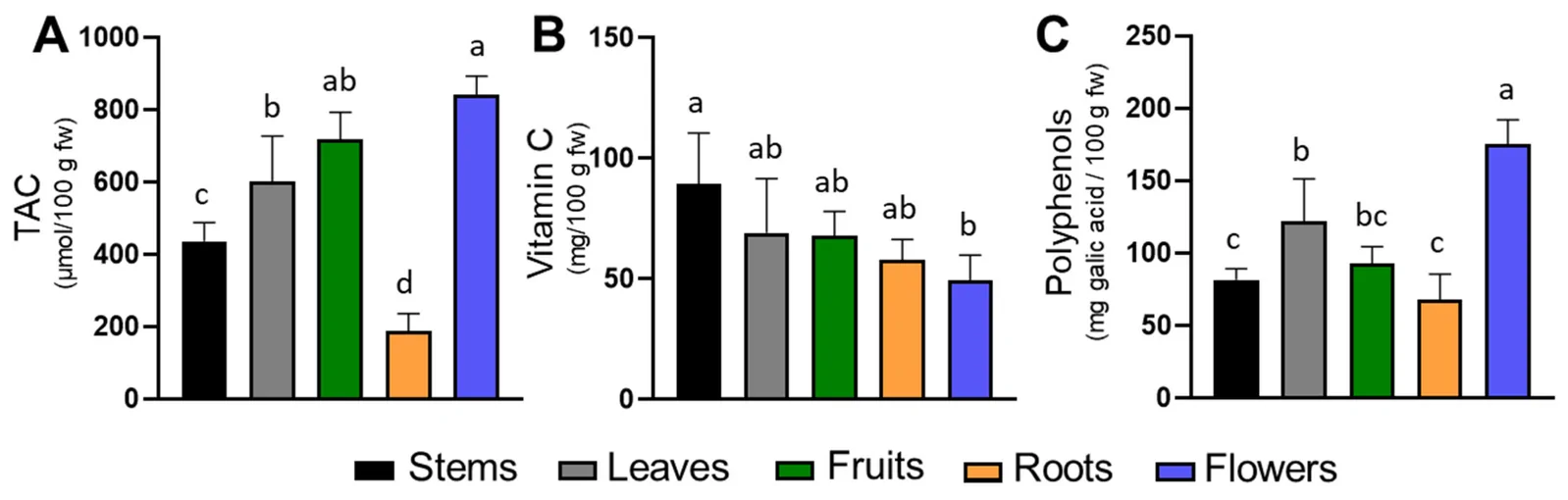
Antioxidant potential of *Cakile maritima* extracts. (**A**) Total antioxidant capacity (TAC) based on the capture of the free radical DPPH, (**B**) vitamin C content, and (**C**) total polyphenols in stems, leaves, fruits, roots and flowers, as indicated. Values are means ± SE. For stems, leaves and fruit samples, *n* = 6; for root and flower samples, *n* = 3. Different letters above the bars indicate statistically significant differences according to one-way ANOVA followed by Tukey’s multiple comparison test (*p* < 0.05).

The antioxidant potential of plants is determined by the activity of antioxidant enzymes and the accumulation of antioxidant compounds able to sequester ROS and other oxidative species [37]. Total antioxidant capacity (TAC) of the different organs of *C. maritima* was determined using the DPPH free radical scavenging assay. Amongst organs, leaves, immature fruits and flowers showed the highest TAC, 601.5, 717.6 and 842.6 µmol/100 g of fw, respectively. These values were significantly higher than those obtained for stems (436.1 µmol/100 g of fw) and roots (186.7 µmol/100 g of fw; Figure 3A).

From a nutritional point of view, however, it is interesting to evaluate the antioxidant capability associated with the accumulation of antioxidant compounds that will be incorporated into the diet, such as vitamin C and total polyphenols. In all vegetative organs and immature fruits, vitamin C accumulation was similar, ranging from 57.7 to 89.2 mg/100 g of fw, whereas flowers presented slightly, though significantly lower concentrations (49.4 mg/100 g of fw; Figure 3B). However, significant differences in total polyphenol accumulation were detected amongst organs, being the highest in flowers (175 mg/100 g of fw), followed by leaves (122 mg/100 g of fw) and immature fruits (92.7 mg/100 g of fw; Figure 3C).

### 3.3. Differential Nutritional and Antioxidant Profiles of Two Morphotypes of Cakile maritima

According to leaf shape, two morphotypes of *C. maritima* have been described, one with pinnatifid leaves and another with entire lamina leaves [12]. Whether leaf morphotype influences the nutritional properties of *C. maritima* was assessed by proximate analysis, mineral composition, and antioxidant potential. On the one hand, fats and fibres were not statistically different in stems, leaves and immature fruits excised from pinnatifid or entire lamina morphotypes (Table 2), whereas slightly but statistically significant differences in dry matter and carbohydrates were observed in stems and fruits. On the other hand, ash percentages showed organ- and morphotype-dependent patterns; whereas stems and immature fruits of the entire lamina morphotype showed higher ash content, they contained slightly but significantly lower ash in their leaves than the pinnatifid morphotype (Table 2). Additionally, total proteins measured within stems, leaves and immature fruits of the entire lamina morphotype were significantly higher than those measured for the pinnatifid morphotypes, as shown in Table 2.

On the other hand, the mineral composition profile of different organs from the entire lamina and pinnatifid leaf morphotypes was analysed (Supplementary Table S2). The entire lamina morphotypes accumulated significantly more calcium in stems and leaves than the pinnatifid leaf morphotype (Supplementary Table S2). The accumulation of potassium and phosphorus, however, was both morphotype- and organ-dependent. Potassium concentration was significantly higher in entire lamina stems and lower in leaves, compared to pinnatifid morphotypes (Supplementary Table S2). Similarly, phosphorus concentration was higher in stems and lower in leaves and fruits in the entire lamina than in the pinnatifid morphotype (Supplementary Table S2).

Finally, the antioxidant potential in the stems, leaves and immature fruits of the two morphotypes was measured. Total antioxidant capacity and total polyphenol content were not significantly different between pinnatifid and entire lamina morphotypes within any organ tested; however, differences in vitamin C were observed (Table 3). Indeed, the entire lamina morphotypes accumulated significantly greater amounts of vitamin C than the pinnatifid morphotype within leaves, whereas it was significantly higher in the stems of the pinnatifid morphotype, compared to the entire leaf morphotype (Table 3). No significant differences were observed in vitamin C concentration between morphotypes in their immature fruits (Table 3).

### 3.4. General Opinion of Potential Consumers of Wild Plants by Online Survey

To study the predisposition of general consumers to incorporate wild resilient plant species, including halophytes, into their diets, an online survey was conducted on an open platform, as described in Materials and Methods (Supplementary Figure S1). A total of 147 participants were randomly recruited and represented a broad range of age groups. Participants were distributed as follows: 21–30 years (25.9%), 31–40 years (10.2%), 41–50 years (38.8%), 51–60 years (19.7%) and over 60 years (5.4%). Moreover, 55.8% of participants were female, 42.9% were male, and 1.4% were non-binary. Finally, the targeted population was in charge of buying groceries always (62.6%) or half the time (27.9%; Supplementary Figure S1A).

**Figure 4:**
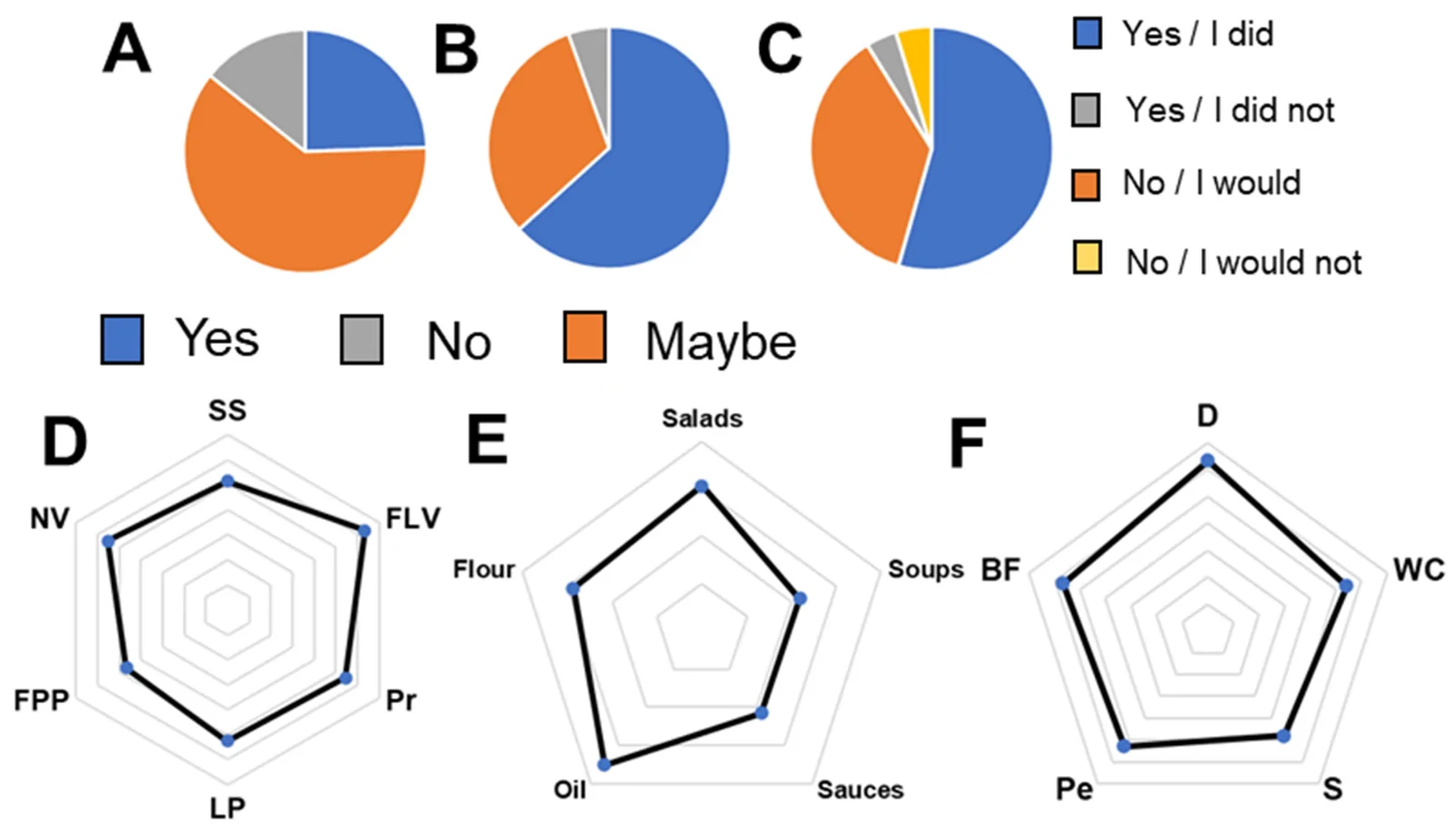
Online survey on food-related habits. (**A**–**C**) Pie charts showing the frequency of responses to the following questions: (**A**) Would you buy a plant-derived food product in a grocery shop? (**B**) Would you order a dish containing a plant-derived food product in a restaurant? (**C**) Do you think it is possible to consume wild plants?/Would you do so? (**D**–**F**) Radar charts obtained from raw data plotted in Supplementary Figure S2A–C, showing the scores obtained for the following questions: (**D**) What is the most appreciated attribute of a plant-derived food product? (**E**) How often do you consume the following items? (**F**) What is the most concerning problem related to agricultural activity? Data conversion into a score-based system as described in Materials and Methods. SS: self-stability; FLV: flavour; Pr: price; LP: local production; FPP: footprint of production; NV: nutritional value; D: drought; WC: water contamination; S: salinity; Pe: pests; BF: benefit for farmers. Non-targeted: 147 participants.

Regarding *acceptance of wild plant consumption*, almost 25% of participants would purchase unknown plant species at a grocery store (Figure 4A), whereas 63.3% would order it in a restaurant (Figure 4B). Only 14.3% and 5.4% of the participants would not be interested in such a food source either in a grocery store or a restaurant, respectively (Figure 4A,B). Since wild plant species are frequently associated with new flavours, the obtained data is coherent with the percentages of people familiarised with so-called innovative, experimental, gourmet or sophisticated cuisine (34%) and those willing to consume it (28.6%; Supplementary Figure S1B). Finally, most participants (91%) indicated that it would be possible to incorporate wild plant species into their diet, and 54% already did so (Figure 4C). Only 5% of the participants would not consume wild plant species (Figure 4C).

Next, regarding the main attributes of food resources to be consumed, the online survey indicated that the most valuable feature was flavour (FLV; Figure 4D), with 52.3% of participants indicating the highest score, followed by the purchasing price (P; 25.8%) and its nutritional value (NV; 25.1%; Figure 4D; Supplementary Figure S2A). Other features related to the ecological cost of production, such as local production (LP), carbon footprint (FPP), and self-stability (SS), were less supported by the participants (Figure 4D; Supplementary Figure S2A), with only 12.9%, 19%, and 19% of the participants selecting them as the most desired aspects, respectively. The participants were also asked about the frequency with which they consume different types of culinary preparations, including salads, flours and flour-based products, oils and their derivatives, sauces, and soups, since *C. maritima* could be used to prepare such types of dishes. According to their responses, oil is the most frequently consumed plant-derived product (Figure 4E), with 85% of participants consuming it daily (Supplementary Figure S2B). Flour-derived products and salads are consumed daily by 40.8% of participants (Figure 4E; Supplementary Figure S2C), whereas soups and sauces are consumed only once per week by most participants (Figure 4E; Supplementary Figure S2C). Amongst plant-derived sauces, wasabi sauce is less frequently consumed than other Asian-style sauces, ketchup, Mexican-style sauces or related mustard sauce (Supplementary Figure S2D,E).

The survey also explored participant perceptions of the main challenges affecting agricultural production by asking them to evaluate the importance of drought, salinity, water pollution, farmers’ benefits and crop pests. Drought was identified as the most important challenge, with 53.7% of respondents assigning it the highest importance score (Figure 4F; Supplementary Figure S2B). By contrast, salinity received the lowest level of concern, with only 14.3% of the participants rating it as a major agricultural problem (Figure 4F; Supplementary Figure S2B). These findings suggest that, despite its increasing impact on agricultural productivity worldwide, soil salinity remains relatively under-recognised by the general public.

### 3.5. Valorisation of Cakile maritima as a Food Source Through Consumer Sensory Testing

**Figure 5:**
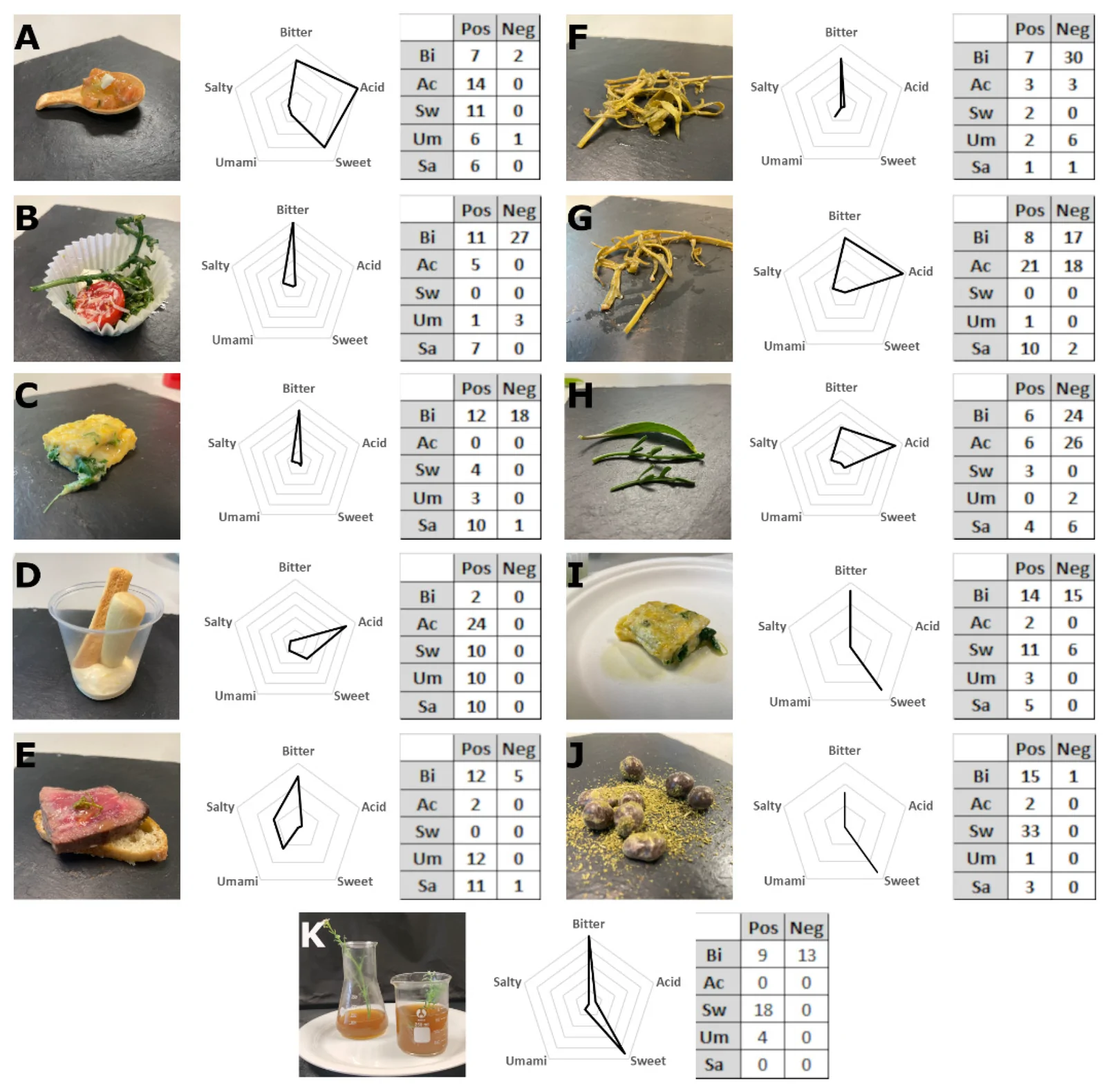
Flavour identification and valorisation of each *Cakile maritima*-based dish tested. For each dish, the following are shown from right to left: a representative image of its overall appearance, a radar chart illustrating the most detectable flavours, and a table reporting the positive and negative effects of the different flavours on consumers’ perception, with their corresponding scores. (**A**) Dish 1: Salmon tartare. (**B**) Dish 2: Caprese salad. (**C**) Dish 3: Salty omelette. (**D**) Dish 4: Mayonnaise elaborated with Salicornia. (**E**) Dish 5: Crystal bread with old beef. (**F**) Dish 6: Salty pickle. (**G**) Dish 7: Sweet pickle. (**H**) Dish 8: Raw tender leaves. (**I**) Dish 9: Sweet omelette. (**J**) Dish 10: Chocolate balls coated with leaf powder. (**K**) Dish 11: Tea presented in lab flask and beaker. Bi: bitter; Ac: acid; Sw: sweet; Um: umami; Sa: salty; Pos: positive valorisation for each identified flavour; Neg: negative valorisation for each identified flavour.

A sensory tasting session comprising 11 dishes was carried out to evaluate the flavour, texture and overall acceptability of *C. maritima* as a culinary ingredient. The participants were asked to indicate their perception of different flavour types (i.e., bitter, acid, sweet, umami and salty) across the 11 dishes and to state whether these flavours were perceived as pleasant (positive) or non-pleasant (negative) within each preparation. Figure 5 shows a representative image of each dish, a radar plot indicating the most perceived flavour, and the positive or negative perception score for each flavour. Overall, the most detected gustatory attribute was bitter, with a score of 158, two-fold higher than acid flavour, the second most identified, with a score of 76 (Table 4). Per dish, bitter flavour was detected in all dishes, with the exception of dish 4 (Figure 5D,J), which corresponded to the control dish that did not include *C. maritima* as an ingredient. For dishes 2, 3, 5 and 6, bitter flavour was by far the most identified one (Figure 5B,C,E,F), whereas for dishes 1, 7, 9, 10 and 11, although detected, it was not the main taste attribute (Figure 5A,G,I–K). Raw tender leaves of *C. maritima*, as the least elaborated dish, showed acidic and bitter as the highest gustatory attributes (Figure 5H).

To assess how different taste attributes were liked by the participants, they were asked whether the flavours detected were pleasant (positive, +) or not (negative, −). Overall, for the bitter taste, the negative perception was higher than the positive one (152 negative score vs. 103 positive score; Table 4), whereas the acidic attribute, the second most perceived, showed the opposite trend (79 positive score vs. 47 negative score; Table 4). The reason why the sum of scores in Table 4 relative to negative and positive perceptions is higher than the detected scores may be due to their differential identification as light or intense. Considering the different dishes, the bitter attribute was perceived as mainly positive in dishes 1 (7 vs. 2; Figure 5A), 5 (12 vs. 5; Figure 5E), and 10 (15 vs. 1; Figure 5J), whereas a mainly unpleasant perception was identified for dishes 2 (11 vs. 27; Figure 5B), 3 (12 vs. 18; Figure 5C), 6 (7 vs. 30; Figure 5F), 7 (8 vs. 17; Figure 5G), and 8 (6 vs. 24; Figure 5H). On the contrary, the acid attribute was indicated to be mostly pleasant for dishes 1 (14 vs. 0; Figure 5A) and 2 (5 vs. 0; Figure 5B), and only dish 8, consisting of raw leaves, was scored mainly as unpleasant (6 vs. 26; Figure 5H).

**Figure 6:**
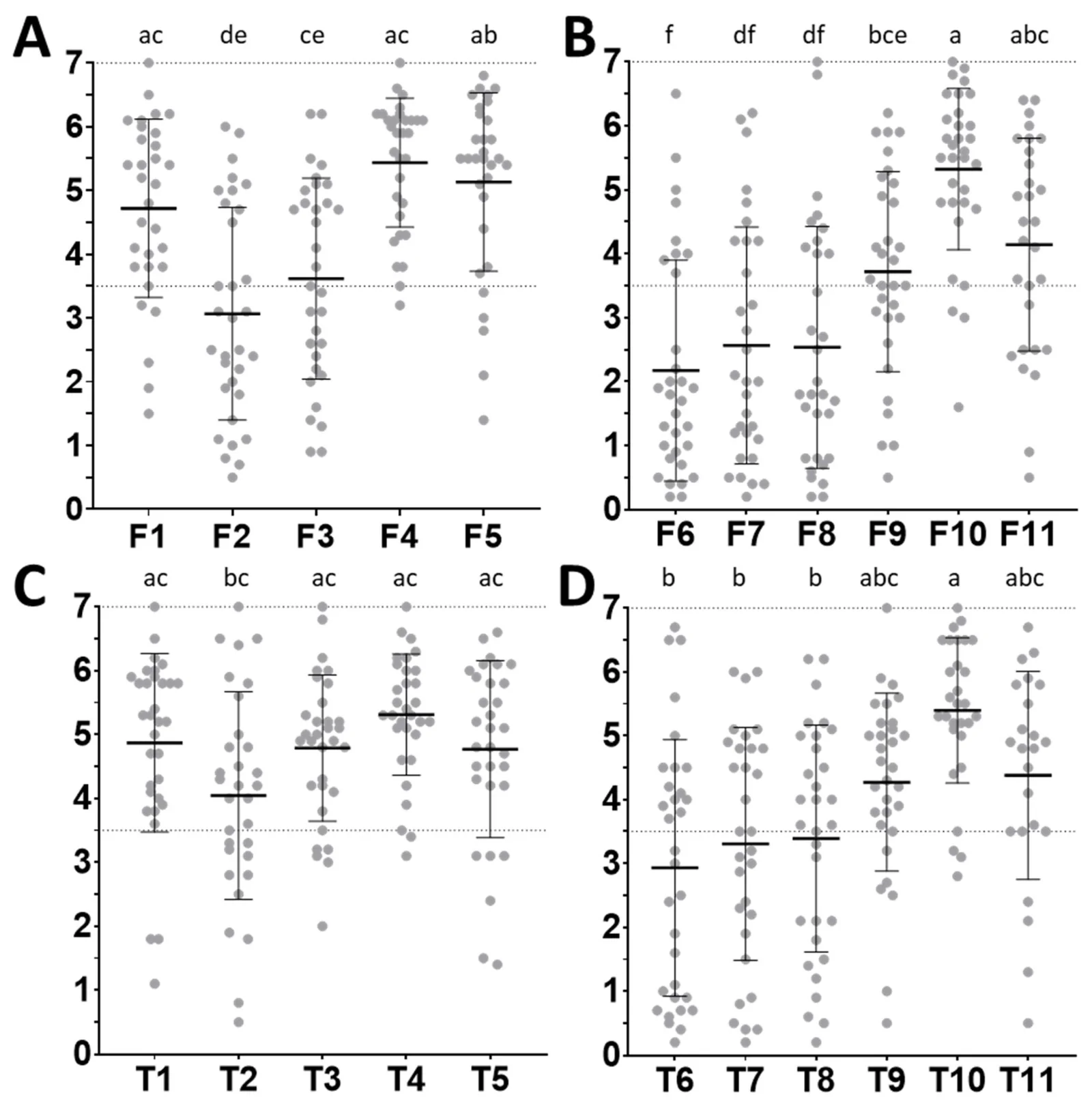
Overall rating of different dishes (1 to 11) elaborated with *C. maritima*, for flavour, F (**A**,**B**) and texture, T (**C**,**D**). Dish 1. Salmon tartare. Dish 2. Caprese salad. Dish 3. Salty omelette. Dish 4. Mayonnaise elaborated with Salicornia. Dish 5. Crystal bread with old beef. Dish 6. Salty pickle. Dish 7. Sweet pickle. Dish 8. Raw tender leaves. Dish 9. Sweet omelette. Dish 10. Chocolate balls coated with leaf powder. Dish 11. Tea. Different letters indicate significant differences between ratings according to Kruskal–Wallis test followed by Dunn’s multiple comparisons test (*p* < 0.05). The dotted line indicates the midpoint of the response scale (3.5), corresponding to half of the maximum score (7), and is included as a reference to distinguish scores above and below the midpoint.

To measure overall acceptance, participants were asked to indicate on a line their appreciation of the taste and texture of the different bites. Participant valorisation was converted into a hedonic scale from 0 to 7 and plotted in a scatterplot indicating average and median values (Figure 6). This graphical representation not only displays the mean, median, and standard error for each dish for both taste and texture but also shows the individual scores of each participant, allowing the variability and dispersion of the data to be readily visualised. According to flavour scores, the most valued dishes were 1, 5 and 10, rated 4.7, 5.1 and 5.3, respectively, as well as the control dish 4, rated 5.3 (Figure 6A,B). A second group was formed by dishes 2, 3, 9 and 11, which scored close to 3.5, whereas the least wanted dishes were 6, 7 and 8 (Figure 6A,B), scored as 2.9, 2.5 and 2.5, respectively. The dendrogram and hierarchical clustering heatmaps (Supplementary Figure S3A) showed that dishes 2, 6, 7 and 8, in which *C. maritima* leaves and stems underwent little or no culinary processing, clustered together and were clearly separated from the remaining preparations. This pattern was consistent with their lower flavour ratings. In contrast, dishes 5, 10 and 11, in which *C. maritima* was more extensively processed, as well as the control dish 4, clustered closely (Supplementary Figure S3A) and represented the most highly rated preparations (Figure 6A,B). A similar trend was observed for texture. The texture of different dishes was overall appreciated, with the exception of the less elaborated (dishes 6, 7 and 8; Figure 6C,D), and, accordingly, these less elaborated dishes clustered together and were clearly separated from the rest of the preparations (Supplementary Figure S3B).

## 4. Discussion

The valorisation of wild edible plants has been increasingly recognised as a key strategy to counteract dietary homogenisation by broadening the biological base of human nutrition and restoring locally adapted food resources. Moreover, in the current scenario of global warming and climate change, wild species naturally adapted to harsh environments represent a suitable alternative food supply, thus enhancing environmental resilience [38]. In this paper, we present a comprehensive nutritional analysis of different organs of the halophyte *Cakile maritima*, together with an assessment of its potential valorisation as a food resource through an online survey and sensory evaluation.

### 4.1. Proximate Analysis of Different Cakile maritima Organs

Leafy plants such as *C. maritima* [16] are characterised by a low dry matter content. In the present study, leaves contained approximately 5% dry matter, comparable to lettuce (ca. 6%) but lower than *Eruca sativa*, *Beta vulgaris* or *Spinacia oleracea* (10–15%; [39]). Although low dry matter is generally associated with reduced postharvest quality owing to moisture loss and continued metabolic activity [40], the high water content of *C. maritima* leaves, consistent with its succulent habit [12], may not necessarily compromise their short-term preservation under refrigerated conditions, as suggested by their maintenance of green colour and turgidity after 10 days of storage [41]. This behaviour may be related to the accumulation of inorganic ions and compatible solutes [20,42], which help maintain cellular turgor after harvest. On the other hand, compared with the close relative white mustard, *C. maritima* leaves contained lower concentrations of carbohydrates and proteins [43].

The nutritional profile of *C. maritima* roots also differed markedly from that of conventional root vegetables. Similar to carrot, *C. maritima* roots contained less than 0.3% fat but exhibited lower carbohydrate and higher fibre, protein and ash contents [44]. Likewise, compared with cassava and sweet potato, whose roots accumulate high levels of carbohydrates but relatively little protein and ash [45,46], *C. maritima* roots displayed a contrasting composition characterised by low carbohydrate and high protein and mineral contents. Consequently, flour or other root-derived foods obtained from *C. maritima* roots may exhibit distinct nutritional properties, potentially contributing to greater dietary diversification. However, further research is needed to confirm this hypothesis.

Edible flowers are increasingly valued in gastronomy for their aesthetic, sensory and nutritional attributes [47,48,49,50]. As previously observed in sea fennel flowers [51], *C. maritima* flowers contained the highest concentrations of carbohydrates and proteins amongst the analysed organs, highlighting their nutritional potential. Further studies addressing aroma characterisation and microbiological safety are nevertheless required before their widespread culinary use [52].

Overall, the proximate composition of *C. maritima* differed markedly between organs and from that of conventional edible species. These findings support the potential of this halophyte as a novel food source that could contribute to greater nutritional diversity in the human diet.

### 4.2. Mineral Profile of Cakile maritima

The mineral composition observed for *C. maritima* in this study differed from previous reports, particularly regarding the high sodium concentrations detected here in the leaves and stems [9]. This discrepancy is likely explained by the moderate salinity of the Albufera lake and surrounding areas [53], where the *C. maritima* plants used in this study were grown until harvested, as salinity promotes sodium uptake and translocation to aerial organs [14,42]. Salinity may also account for the differences observed in other mineral elements. According to the well-documented antagonism between sodium and potassium under saline conditions [13], potassium concentration remained low compared to previous reports [18]. Calcium concentrations in leaves and stems were three- to six-fold higher than previously reported [9,15]; however, the relation between higher calcium concentration and salinity is not clearly established in *C. maritima* [13,18,41], as it is in other halophytes [54].

Magnesium levels were also generally higher than those reported by [9], although lower than values described by [15]. On the contrary, micronutrient concentrations were largely consistent across different studies. Collectively, these findings indicate that the mineral composition of *C. maritima* is strongly influenced by environmental conditions, including salinity, as well as the plant developmental stage, and that these factors should be considered when evaluating its nutritional potential.

Marked differences in mineral distribution were observed amongst organs. Whereas the halophyte *Suaeda salsa* accumulates calcium, magnesium and phosphorus mainly in leaves, and iron, zinc, manganese and copper in roots [55], *C. maritima* accumulated most minerals preferentially in the aerial organs, especially in leaves.

From a dietary perspective, the nutritional relevance of *C. maritima* varies substantially depending on the plant organ consumed, and this fact should be considered for specific diets. Leaves and, to a lesser extent, stems were the most relevant organs in terms of their mineral composition and abundance. Leaves were especially rich in calcium; thus, their consumption could make a meaningful contribution to dietary calcium intake. Similarly, *C. maritima* leaves may significantly contribute to the uptake of other elements, such as Mg and K. On the other hand, although elevated sodium concentrations observed in these organs may limit the nutritional value, the levels measured in *C. maritima* remained below the maximum intake recommended by the European Food Safety Authority (EFSA) [56] and were considerably lower than those reported for other edible halophytes [51]. Nevertheless, the relatively high concentration of sodium observed in leaves and stems should be taken into account when considering their incorporation into sodium-restricted diets. Sodium concentration in other organs, however, remains low.

Phosphorus concentrations were unexpectedly high across different organs analysed compared with previous reports in *C. maritima* [18] and other halophytes [51,57]. This observation may be partially related to the high phosphorus inputs historically reported in soils and waters of Albufera lake [58], where the plants were grown, and may reflect an efficient capacity for phosphorus uptake and accumulation, potentially associated with its adaptation to saline environments. Nevertheless, given the unusually high concentration detected, further confirmation using complementary analytical techniques and controlled cultivation conditions would be advisable to establish whether these values are conserved in *C. maritima* tissues. Notably, flowers were particularly rich in phosphorus, suggesting that they may provide additional nutritional value. However, because phosphorus bioavailability from plants largely depends on its chemical form, which was not determined in the present study, further analyses are required to establish its nutritional relevance [59]. The contribution of *C. maritima* consumption to dietary intakes of other micronutrients, such as Fe, Cu and Zn, is rather limited.

Nevertheless, the potential accumulation of toxic elements should also be considered when assessing its suitability as a food resource. *Cakile maritima* has been shown to accumulate Cd when growing in contaminated soils [60]. However, Cd concentrations in the present study were below the limit of detection. Arsenic (As), another potentially toxic element, was not quantified in this study, and further research may be valuable in this regard. Nevertheless, previous in vitro assays using mammalian cell cultures indicated low or no toxicity of *C. maritima* extracts [61].

Overall, *C. maritima* cannot be regarded as a food with a single specific dietary purpose. Rather, its nutritional relevance varies depending on the plant organ consumed. Given its expected use primarily as a complementary ingredient rather than as a staple vegetable, its contribution to overall dietary mineral intake will also depend on the amount incorporated into culinary preparations. Further studies addressing the bioavailability of these elements are therefore warranted to fully establish the nutritional value and dietary relevance of the different edible organs of *C. maritima*.

### 4.3. Antioxidant Potential of Cakile maritima

The antioxidant potential estimated in the present study by DPPH radical scavenging activity was comparable to that previously reported for *C. maritima* [61] and for common edible species with recognised antioxidant properties, such as *Asparagus acutifolius* [62]. Notably, the antioxidant potential of *C. maritima* was higher than that reported for leaves and stems of other edible halophytes, including *Sarcocornia ambigua* [63] and *Tecticornia* sp. [64].

Polyphenol concentrations were slightly lower than those previously quantified by liquid chromatography in *C. maritima* leaves [61]. Compared with other edible halophytes, however, polyphenol levels were substantially lower than those reported for aqueous extracts of different *S. ambigua* biotypes [65], but higher than those reported for *Tecticornia* sp. [64]. Vitamin C concentrations were generally comparable to those previously reported for wild populations of *C. maritima* [15,18,66] and *A. acutifolius* [62], and slightly higher than those reported for other edible halophytes [64,65].

Interestingly, vitamin C levels in naturally growing *C. maritima* populations were markedly lower than those observed for plants cultivated in pots under greenhouse conditions [67]. This difference suggests that cultivation conditions may substantially influence the metabolic profile of *C. maritima*, as reported for other plant species [68,69]. Since most physiological and biochemical studies of *C. maritima* have been conducted using potted plants under controlled greenhouse conditions, the accumulation of stress-related metabolites, including ascorbate, glutathione, proline and other antioxidants, may not fully reflect that occurring in wild populations. Similar differences between greenhouse- and field-grown plants have recently been reported for the halophyte *Plantago coronopus*, which exhibited higher antioxidant activity and greater polyphenol and flavonoid accumulation under natural conditions [70].

It should be noted that the present study focused on polyphenols and vitamin C as nutritionally relevant non-enzymatic antioxidant compounds. However, the antioxidant capacity measured by DPPH may also reflect the contribution of other non-enzymatic antioxidants, such as glutathione, carotenoids and tocopherols, as well as antioxidant enzymes, which have been previously described for *C. maritima* [19,71,72]. Therefore, the relatively high antioxidant capacity observed in *C. maritima* cannot be attributed exclusively to the accumulation of polyphenols and vitamin C. Further research should therefore investigate a broader range of antioxidant compounds and their bioavailability, particularly those with established nutritional relevance, to better characterise the contribution of the antioxidant profile of *C. maritima* to its potential health-promoting value as a food resource.

### 4.4. Differences in Nutritional Properties of Two Morphotypes of Cakile maritima

Proximate composition is widely used to evaluate the nutritional quality of edible plants [73]. Although both *C. maritima* morphotypes exhibited similar energy-related traits, with no differences in carbohydrate or fat content, significant differences were detected in protein and ash content. Variation in ash content, particularly in stems, exceeded that reported for soybean cultivars [74] and cassava [46], whereas differences in protein content were modest compared with those induced by nitrogen fertilisation in leafy vegetables [39]. These findings indicate that morphotype selection may influence the protein and mineral composition of *C. maritima*; however, its nutritional relevance should be considered together with agronomic performance and stress tolerance.

Higher antioxidant activity has been previously reported in the pinnatifid morphotype than in the entire lamina morphotype [18], whereas we have not detected significant differences between morphotypes. This discrepancy may reflect differences in the analysed material, as previous studies evaluated whole aerial organs [18], whereas we analysed leaves and stems separately. Nevertheless, in our experiments, the pinnatifid morphotypes consistently showed a tendency towards higher antioxidant capacity, although differences were not statistically significant. Consistent with their similar antioxidant capacity, both morphotypes accumulated comparable levels of polyphenols, in agreement with previous observations [18]. Interestingly, we observed an opposite vitamin C accumulation pattern between stems and leaves in both morphotypes. Whether this observation is related to morphotype differences in vitamin C transport or selective accumulation in photosynthetic organs needs further research.

### 4.5. Consumer Perception and Willingness to Consume Wild Edible Plants

Citizen-science approaches, including online surveys, are increasingly used to investigate consumer preferences, food innovation, sustainable food systems and the acceptance of wild edible plants [75,76,77,78,79,80]. Consistent with current trends, our survey indicates growing interest in wild edible plants, driven by cultural exchange, the search for novel flavours and culinary experiences, and the increasing demand for resilient crops adapted to adverse environmental conditions [81,82,83]. This is in agreement with the steady increase in scientific research on wild edible plants over the last decade [84].

Flavour emerged as the most important characteristic influencing consumer preferences, consistent with previous studies identifying flavour, appearance, texture and aroma as the main determinants of food acceptance [80,85]. Although environmental awareness is increasingly shaping consumer choices and sustainable production has been recognised as an important factor in the acceptance of wild edible plants [80,86,87], local production and carbon footprint were amongst the least valued attributes in the present survey.

Finally, respondents identified drought as the principal challenge for agricultural production, whereas salinity was perceived as a comparatively minor concern despite representing a major agronomic constraint in many regions [88]. These findings suggest that saline agriculture is not yet recognised by the surveyed population as a relevant adaptation strategy. Although this perception may reflect the predominantly Spanish Mediterranean origin of the respondents, where salinity is not yet widely regarded as a major agricultural problem, projections indicate that soil salinisation will intensify during the coming decades, particularly in Mediterranean coastal areas, increasing its importance for future agricultural production [2].

### 4.6. Sensory Perception and Gastronomic Potential of Cakile maritima

The sensory evaluation conducted in the present study identified bitterness as the predominant flavour attribute in most dishes containing *C. maritima*. This pronounced bitterness is a common feature of many wild edible plants and represents a distinctive sensory characteristic that differentiates them from conventional vegetables [89]. Similar observations have been reported for other wild edible species, in which bitterness was the dominant sensory attribute and its intensity was influenced by cultivation conditions, with greenhouse- or pot-grown plants generally perceived as less bitter than wild populations [30]. Likewise, bitterness has been recognised as a recurrent sensory trait in several native New Zealand edible plants, where it plays an important role in consumer acceptance [90].

Although this flavour profile may appeal to consumers seeking novel culinary experiences, it is also likely to limit the broader acceptance of *C. maritima*. In the present study, bitterness was positively perceived by fewer than half of the participants, suggesting that this species may be more attractive to a specific consumer niche than to the general public. Consequently, while its sensory profile may restrict its incorporation into mainstream diets, it could also be a distinctive attribute for gourmet cuisine and for markets focused on traditional or wild edible foods.

Nevertheless, bitterness should not necessarily be regarded solely as a limiting factor, as suitable culinary approaches may mitigate or complement this flavour. Indeed, the results of the testing event presented in this study suggest that combinations with fatty ingredients or sweet flavours may improve the acceptability of bitterness, as indicated by the favourable scores obtained in dishes 5 and 10, respectively. Thus, the development of recipes specifically designed to balance bitterness with other sensory components could improve consumer acceptance and facilitate the incorporation of *C. maritima* into a wider range of food products. Such gastronomic innovation may ultimately help expand its market potential while maintaining its unique sensory identity.

Some limitations of the consumer survey and sensory evaluation should be considered. The use of snowball sampling may have introduced selection bias, as participants were recruited through personal and social networks and therefore may share similar sociodemographic characteristics, attitudes or food preferences. Consequently, the sample composition may not fully represent the broader population, and the survey results should not be interpreted as representative of consumer preferences at the population level. In addition, preparing dishes containing *C. maritima* was relatively complex and required the collaboration of a professional chef. This complexity limited the number of dishes and participants that could be included in the tasting event, preventing a larger and more representative sensory assessment. Nevertheless, sensory evaluations of similar wild or underutilised food resources have also been conducted with relatively small numbers of participants [91,92,93]. Despite these limitations, the online survey and sensory evaluation presented in this work offer an exploratory assessment of consumer perceptions and the gastronomic potential of the wild halophyte *C. maritima*, providing a basis for future studies involving larger and more representative samples.

### 4.7. Future Perspectives

The results presented in this study highlight the potential of *C. maritima* as a valuable food resource and further support the interest in this species beyond its ecological significance. In addition to its nutritional value, *C. maritima* represents a promising species for saline agriculture and land restoration strategies. As a salt-accumulating halophyte [14], it may contribute to the rehabilitation of salt-affected soils through biomass production, salt removal and improvements in soil properties, thereby supporting the recovery of degraded environments [8]. However, wild edible species, including the halophyte *C. maritima*, should not be viewed as a substitute for conventional crops, but rather as a complementary species that can be integrated into agricultural systems established on marginal or salinised lands. Such integration could enhance the productive use of these areas, increase agroecosystem resilience and reduce dependence on freshwater resources. Consequently, the cultivation of halophytes is increasingly regarded as a valuable component of climate-resilient and nature-based agricultural practices. This approach aligns with emerging concepts of climate-smart and nature-based agriculture [94,95].

## 5. Conclusions

*Cakile maritima* exhibited a favourable nutritional profile characterised by a relatively high water content, elevated root fibre, high ash content in the leaves, and increased protein concentrations in leaves and flowers, together with low carbohydrate and fat contents. The species also showed a rich and diverse mineral composition, with particularly high sodium levels in the leaves, although these remained within recommended dietary limits. Furthermore, the aerial organs were an excellent source of vitamin C. Despite some differences between the two morphotypes, their nutritional composition and antioxidant properties were broadly comparable. The sensory evaluation revealed a positive predisposition towards consuming this wild halophyte. To the best of our knowledge, this is the first study to report a structured tasting event based on elaborated dishes incorporating a wild halophyte, accompanied by a consumer acceptance assessment. These findings support the potential of *C. maritima* as a sustainable and nutritious food resource with promising gastronomic applications.

## Figures and Tables

**Table 1:** Macro- and microelement quantification in different *Cakile maritima* organs, as indicated, in milligrams of the corresponding element per 100 g of dry weight (mg 100 g dw^−1^). Values indicate means ± SE. For stems, leaves and fruit samples, *n* = 6; for root and flower samples, *n* = 3. Different letters in each column indicate statistically significant differences according to one-way ANOVA followed by Tukey’s multiple comparison test (*p* < 0.05).

	**Na**	**Ca**	**K**	**Mg**	
Stems	378.73 ± 10.53 ^d^	938.99 ± 52.12 ^b^	1905.62 ± 122.85 ^b^	66.31 ± 11.39 ^c^	
Leaves	680.81 ± 19.03 ^a^	2530.91 ± 68.06 ^a^	2743.27 ± 182.77 ^a^	362.08 ± 5.48 ^a^	
Fruits	177.58 ± 9.15 ^bc^	903.99 ± 11.48 ^b^	1435.92 ± 19.80 ^b^	110.75 ± 2.37 ^b^	
Roots	229.90 ± 4.62 ^b^	263.83 ± 20.21 ^c^	2007.16 ± 36.65 ^b^	nd ^d^	
Flowers	136.69 ± 15.52 ^c^	705.66 ± 43.81 ^b^	1613.06 ± 86.90 ^b^	127.10 ± 16.38 ^b^	
	**P**	**Fe**	**Cu**	**Zn**	**B**
Stems	553.71 ± 19.19 ^e^	3.32 ± 0.69 ^a^	0.29 ± 0.02 ^a^	1.56 ± 0.11 ^a^	0.95 ± 0.06 ^a^
Leaves	1731.18 ± 79.60 ^b^	2.68 ± 1.45 ^a^	0.16 ± 0.04 ^b^	0.49 ± 0.18 ^b^	0.6 ± 0.18 ^ab^
Fruits	1082.51 ± 20.13 ^c^	0.06 ± 0.01 ^a^	0.06 ± 0.00 ^b^	0.12 ± 0.01 ^b^	0.11 ± 0.01 ^c^
Roots	910.23 ± 21.53 ^cd^	0.16 ± 0.02 ^a^	0.07 ± 0.00 ^b^	0.27 ± 0.03 ^b^	0.23 ± 0.01 ^bc^
Flowers	2118.37 ± 166.41 ^a^	0.10 ± 0.02 ^a^	0.06 ± 0.00 ^b^	0.23 ± 0.03 ^b^	0.27 ± 0.04 ^bc^
	**Pb**	**Mn**	**Mo**	**Se**	**Hg**
Stems	0.07 ± 0.01 ^a^	0.59 ± 0.02 ^a^	0.02 ± 0.01 ^a^	0.03 ± 0.01 ^a^	0.04 ± 0.00 ^a^
Leaves	0.11 ± 0.01 ^a^	0.63 ± 0.34 ^a^	0.01 ± 0.00 ^a^	0.04 ± 0.01 ^a^	0.03 ± 0.00 ^b^
Fruits	0.09 ± 0.02 ^a^	0.03 ± 0.00 ^a^	0.01 ± 0.00 ^a^	0.04 ± 0.01 ^a^	0.03 ± 0.00 ^b^
Roots	0.13 ± 0.03 ^a^	0.03 ± 0.00 ^a^	0.02 ± 0.00 ^a^	0.03 ± 0.01 ^a^	0.02 ± 0.00 ^b^
Flowers	0.12 ± 0.00 ^a^	0.03 ± 0.00 ^a^	0.01 ± 0.00 ^a^	0.06 ± 0.00 ^a^	0.02 ± 0.00 ^b^

**Table 2:** Proximate analysis of different edible organs of pinnatifid and entire lamina morphotypes of *Cakile maritima*. Values indicate means ± SE (*n* = 3) and are presented as % of dry weight (dw; ashes, total protein, and fats) or fresh weight (fw; dry matter, fibre, and carbohydrates). Asterisks indicate statistically significant differences amongst leaf morphotypes according to pairwise *t*-tests: *p* < 0.05 (*), *p* < 0.01 (**), *p* < 0.001 (***).

		Dry Matter	Ashes	Fibre	Fats	Total Protein	Carbohydrates
Stems	Pinnatifid	15.02 ± 0.09	7.6 ± 0.11	7.05 ± 0.04	1.89 ± 0.15	4.30 ± 0.11	5.89 ± 0.10
	Entire Lamina	12.11 ± 0.88 *	10.15 ± 0.23 ***	5.67 ± 0.42	1.77 ± 0.07	5.32 ± 0.01 ***	4.35 ± 0.30 **
Leaves	Pinnatifid	4.08 ± 0.27	21.78 ± 0.10	0.61 ± 0.08	4.69 ± 0.35	12.60 ± 0.30	1.88 ± 0.14
	Entire Lamina	4.92 ± 0.23	20.45 ± 0.25 **	1.21 ± 0.15	2.68 ± 1.19	14.26 ± 0.14 **	1.86 ± 0.09
Fruits	Pinnatifid	9.38 ± 0.56	7.36 ± 0.04	2.69 ± 0.24	2.34 ± 0.09	7.35 ± 0.16	5.10 ± 0.25
	Entire Lamina	6.57 ± 0.47 *	8.81 ± 0.19 **	2.30 ± 0.10	1.80 ± 0.25	8.16 ± 0.11 ***	2.89 ± 0.32 *

**Table 3:** Antioxidant profile of different edible organs of pinnatifid and entire lamina morphotypes of *Cakile maritima*. TAC (µmol/100 g fw); Vitamin C and Polyphenols (mg/100 g fw). Values indicate means ± SE (*n* = 3). Asterisks indicate statistically significant differences amongst leaf morphotypes according to pairwise *t*-tests: *p* < 0.05 (*), *p* < 0.01 (**).

		TAC	Vitamin C	Polyphenols
Stems	Pinnatifid	463.98 ± 24.51	107.82 ± 1.81	86.12 ± 4.14
	Entire Lamina	408.30 ± 29.42	70.53 ± 5.19 **	76.88 ± 3.60
Leaves	Pinnatifid	628.48 ± 103.59	47.15 ± 28.69	126.37 ± 23.14
	Entire Lamina	574.50 ± 40.16	83.78 ± 7.34 *	117.59 ± 12.28
Fruits	Pinnatifid	711.05 ± 64.32	68.47 ± 3.23	89.31 ± 9.03
	Entire Lamina	724.25 ± 26.36	66.89 ± 8.77	96.01 ± 4.69

**Table 4:** Overall score of detected flavours and whether these were considered pleasant (positive) or unpleasant (negative) amongst the 11 dishes prepared in the sensory tasting event. Scores for flavours (detected, negative and positive) were obtained accounting for the number of times identified by participants along the 11-dish degustation and the intensity (light or intense), as described in Materials and Methods.

	Bitter	Acid	Sweet	Umami	Salty
Detected	158	76	51	19	24
Negative	152	47	6	12	11
Positive	103	79	92	43	67

## Data Availability

The original contributions presented in this study are included in the article/Supplementary Material. Further inquiries can be directed to the corresponding author.

## Supplementary Materials

The following supporting information can be downloaded at: https://www.mdpi.com/article/10.3390/foods15183261/s1, Figure S1. Online survey on food-related habits I. (A–C) Pie charts showing the distribution of responses to the following questions: (A) Do you do the grocery shopping in your household? (B) Do you consume alternative or gourmet cuisine? (C) Are you familiarized with the wild species *Cakile maritima*? (D) Do you recognise this species in the image shown in (E)? (E) Image showing the overall appearance of *Cakile maritima* (source: Wikipedia). Non-targeted 147 participants. Figure S2. Online survey on food-related habits II. (A,B). Bar charts showing the number of participants that indicated the importance of (A) different characteristics of a plant-derived food source, (B) and the main concerns and challenges of agriculture. (C,D) Bar charts showing consumers preferences for (C) different types of dishes and (D) sauces elaborated with plant-derived food sources. (E) Data plotted in (D) is represented in a radar chart. SS: Shelf-Stability; FLV: flavour; P: Price; LP: Local Production; FPP: Footprint of Production; NV: Nutritional Value; D: Drought; WC: Water Contamination; S: Salinity; P: Price; BF: benefit for farmers. Non-targeted 147 participants. Figure S3. Hierarchical clustering heatmap of the dishes based on individual ratings. Hierarchical clustering heatmap of the dishes based on individual (A) flavour and (B) texture ratings. Rows represent dishes and columns represent participants. Colours indicate scores on a 0–7 scale, from low (blue) to high (red). Grey indicates missing values. Dendrogram groups dishes with similar (A) flavour- or (B) texture-rating patters. Table S1. Macro and microelement quantification in different organs of *Cakile maritima*, as indicated, in milligrams of the corresponding element per 100 grams of fresh weight (mg 100 g fw^−1^). Values indicate means ± SE. For stems, leaves and fruit samples, n = 6; for root and flower samples, n = 3. Different letters in each column indicate statistically significant differences according to one-way ANOVA followed by Tukey’s multiple comparison test (*p* < 0.05). Table S2. Macro and microelement quantification in different organs excised from different leaf morphotypes (pinnatifid and entire lamina) of *Cakile maritima*, as indicated, in milligrams of the corresponding element per 100 grams of dry weight (mg 100 g dw^−1^). Values represent means ± SE (n = 3). Asterisks indicate statistically significant differences among leaf morphotypes according to pairwise *t*-tests (*p* < 0.05).
