# Supplementary material for: Valorisation of the Halophyte Cakile maritima as a Food Resource for Human Consumption
Source: Foods. 2026 Sep 15;15(18):3261. doi: 10.3390/foods15183261 (PMC13606383; doi:10.3390/foods15183261)
Supplement: Supplementary file 1 [file foods-15-03261-s001.zip › foods-4504753-supplementary.pdf]

Supplementary Materials:

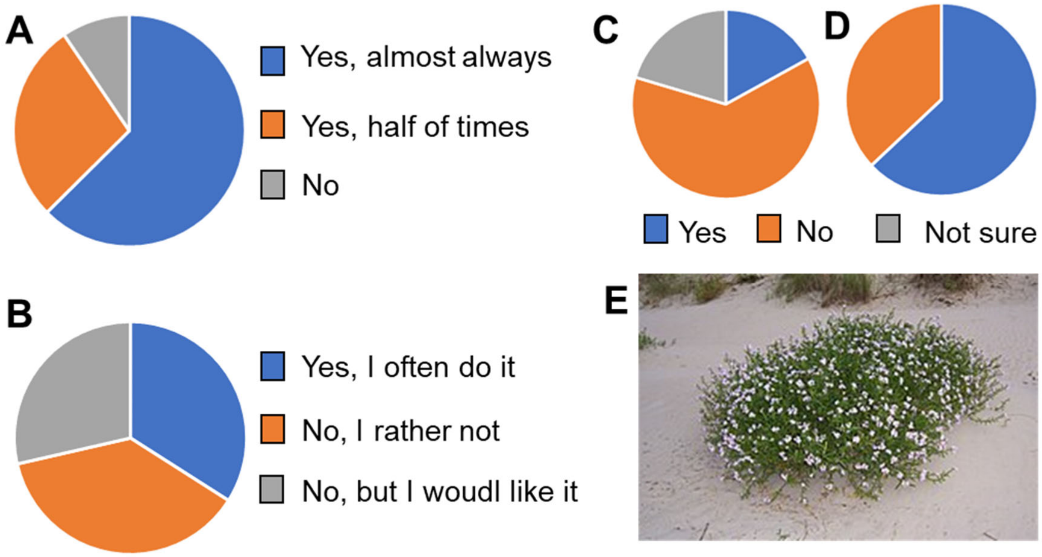

Supplementary Figure S1. Online survey on food-related habits I. (A–C) Pie charts showing the distribution of responses to the following questions: (A) Do you do the grocery shopping in your household? (B) Do you consume alternative or gourmet cuisine? (C) Are you familiarized with the wild species *Cakile maritima*? (D) Do you recognise this species in the image shown in (E)? (E) Image showing the overall appearance of *Cakile maritima* (source: Wikipedia). Non-targeted 147 participants.

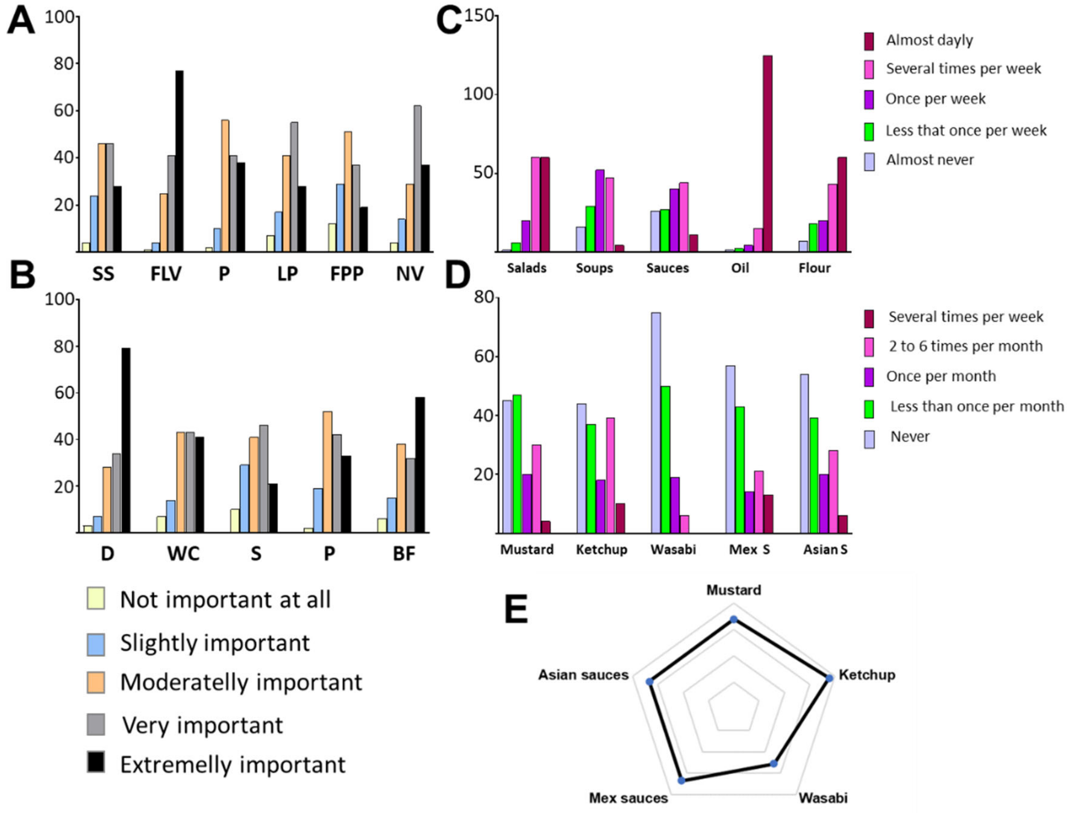

Supplementary Figure S2. Online survey on food-related habits II. (A, B). Bar charts showing the number of participants that indicated the importance of (A) different characteristics of a plant-derived food source, (B) and the main concerns and challenges of agriculture. (C, D) Bar charts showing consumers preferences for (C) different types of dishes and (D) sauces elaborated with plant-derived food sources. (E) Data plotted in (D) is represented in a radar chart. SS: Shelf-Stability; FLV: flavour; P: Price; LP: Local Production; FPP: Footprint of Production; NV: Nutritional Value; D: Drought; WC: Water Contamination; S: Salinity; P: Price; BF: benefit for farmers. Non-targeted 147 participants.

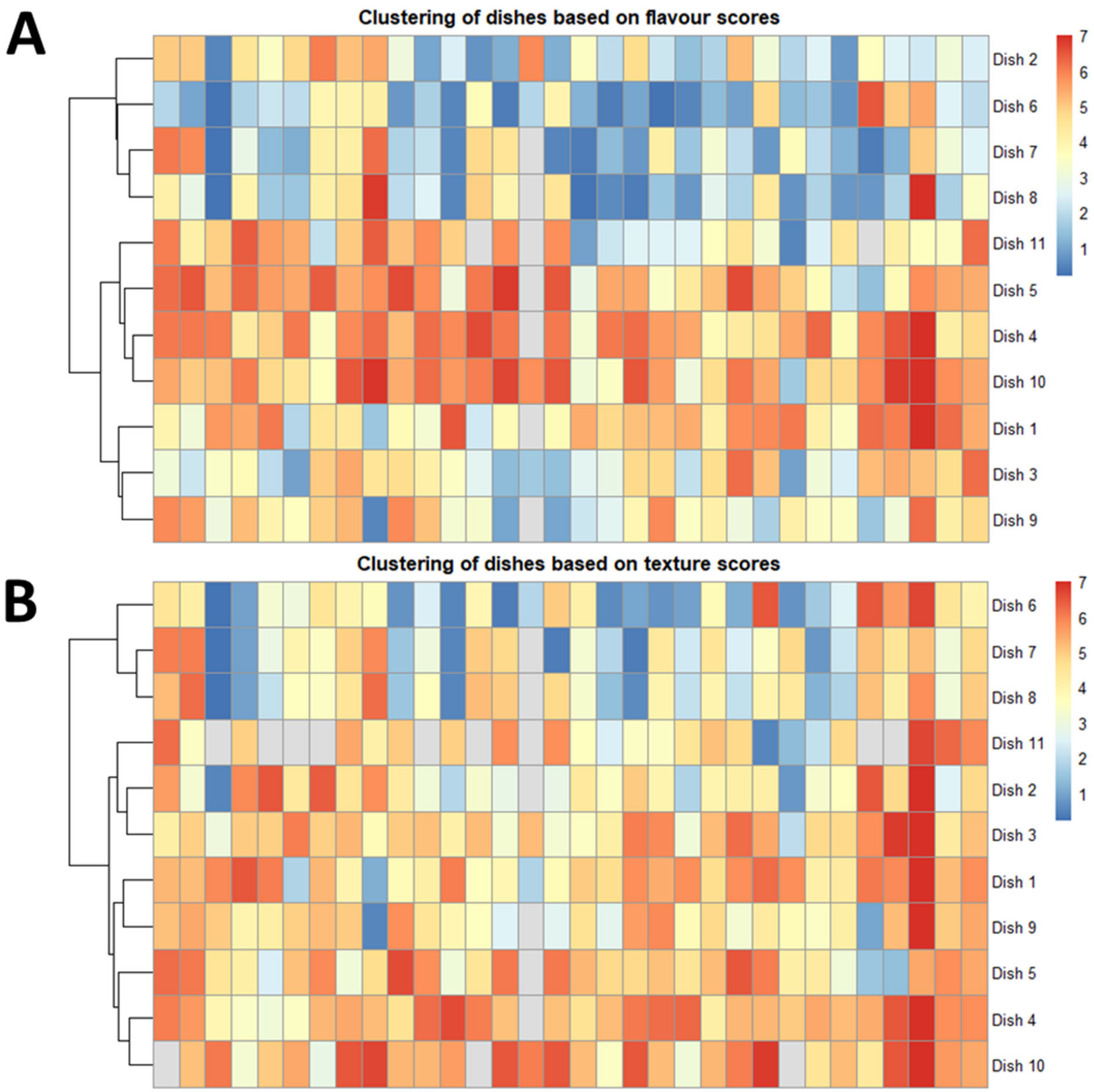

Supplementary Figure S3. Hierarchical clustering heatmap of the dishes based on individual ratings. Hierarchical clustering heatmap of the dishes based on individual (A) flavour and (B) texture ratings. Rows represent dishes and columns represent participants. Colours indicate scores on a 0-7 scale, from low (blue) to high (red). Grey indicates missing values. Dendrogram groups dishes with similar (A) flavour- or (B) texture-rating patters.

Supplementary Table S1. Macro and microelement quantification in different organs of *Cakile maritima*, as indicated, in milligrams of the corresponding element per 100 grams of fresh weight (mg 100 g fw -1). Values indicate means ± SE. For stems, leaves and fruit samples, *n* = 6; for root and flower samples, *n* = 3. Different letters in each column indicate statistically significant differences according to one-way ANOVA followed by Tukey's multiple comparison test ($p < 0.05$).

	Na	Ca	K	Mg
Stems	51.37 ± 3.32 ^{a}	125.83 ± 5.19 ^{ab}	254.47 ± 9.66 ^{a}	8.65 ± 1.22 ^{b}
Leaves	59.47 ± 15.03 ^{a}	220.80 ± 56.96 ^{a}	216.28 ± 41.01 ^{a}	31.10 ± 7.61 ^{a}
Fruits	12.43 ± 2.21 ^{b}	63.66 ± 10.62 ^{b}	101.18 ± 16.92 ^{b}	7.81 ± 1.32 ^{b}
Roots	23.23 ± 1.51 ^{ab}	26.50 ± 1.83 ^{b}	201.95 ± 7.04 ^{ab}	nd ^{c}
Flowers	19.67 ± 2.23 ^{ab}	101.53 ± 6.11 ^{ab}	232.10 ± 12.11 ^{a}	18.28 ± 2.32 ^{ab}

	P	Fe	Cu	Zn	B
Stems	75.11 ± 2.73 ^{b}	0.45 ± 0,10 ^{a}	0.04 ± 0.00 ^{a}	0.21 ± 0.00 ^{a}	0.13 ± 0.00 ^{a}
Leaves	77.96 ± 16.17 ^{b}	0.13 ± 0.05 ^{b}	0.01 ± 0.00 ^{b}	0.03 ± 0.00 ^{b}	0.04 ± 0.00 ^{b}
Fruits	86.38 ± 14.82 ^{b}	0.00 ± 0.00 ^{b}	0.00 ± 0.00 ^{b}	0.01 ± 0.00 ^{b}	0.01 ± 0.00 ^{b}
Roots	77.13 ± 2.65 ^{b}	0.02 ± 0.00 ^{b}	0.01 ± 0.00 ^{b}	0.03 ± 0.00 ^{b}	0.02 ± 0.00 ^{b}
Flowers	256.73 ± 19.72 ^{a}	0.02 ± 0.00 ^{b}	0.01 ± 0.00 ^{b}	0.03 ± 0.00 ^{b}	0.04 ± 0.00 ^{b}

	Pb	Mn	Mo	Se	Hg
Stems	0.009 ± 0.001 ^{ab}	0.081 ± 0.007 ^{b}	0.003 ± 0.002 ^{a}	0.005 ± 0.002 ^{ab}	0.006 ± 0.001 ^{a}
Leaves	0.009 ± 0.003 ^{ab}	0.031 ± 0.120 ^{a}	0.001 ± 0.000 ^{a}	0.003 ± 0.000 ^{b}	0.002 ± 0.001 ^{b}
Fruits	0.006 ± 0.002 ^{b}	0.002 ± 0.001 ^{b}	0.001 ± 0.000 ^{a}	0.002 ± 0.001 ^{b}	0.002 ± 0.001 ^{b}
Roots	0.013 ± 0.002 ^{ab}	0.003 ± 0.000 ^{ab}	0.001 ± 0.000 ^{a}	0.003 ± 0.001 ^{ab}	0.002 ± 0.000 ^{b}
Flowers	0.018 ± 0.000 ^{a}	0.004 ± 0.000 ^{ab}	0.001 ± 0.000 ^{a}	0.009 ± 0.002 ^{ab}	0.003 ± 0.000 ^{b}

Supplementary Table S2. Macro and microelement quantification in different organs excised from different leaf morphotypes (pinnatifid and entire lamina) of *Cakile maritima*, as indicated, in milligrams of the corresponding element per 100 grams of dry weight (mg 100 g dw $^{-1}$). Values represent means ± SE (*n* = 3). Asterisks indicate statistically significant differences among leaf morphotypes according to pairwise t-tests ($p < 0.05$).

		Na	Ca	K	Mg
Stems	Pinnatifid	376.88 ± 23.42	824.78 ± 4.29	1631.93 ± 19.02	41.20 ± 2.00
	Entire Lamina	380.58 ± 1.48	1053.20 ± 22.80 ***	2179.31 ± 13.95 ****	91.43 ± 3.75 ***
Leaves	Pinnatifid	650.08 ± 23.17	2416.43 ± 56.71	3151.43 ± 20.02	357.87 ± 2.00
	Entire Lamina	711.53 ± 18.16	2645.38 ± 82.73 *	2335.12 ± 5.07 ****	366.30 ± 11.33
Fruits	Pinnatifid	175.42 ± 20.33	925.71 ± 7.95	1470.65 ± 14.97	114.27 ± 2.70
	Entire Lamina	179.74 ± 0.54	882.26 ± 11.13 *	1401.19 ± 23.02	107.24 ± 2.91

		P	Fe	Cu	Zn	B
Stems	Pinnatifid	512.50 ± 7.53	2.59 ± 0.12	0.31 ± 0.03	1.78 ± 0.11	1.05 ± 0.10
	Entire Lamina	594.92 ± 9.22 **	4.05 ± 1.35	0.27 ± 0.01	1.33 ± 0.01 *	0.86 ± 0.02
Leaves	Pinnatifid	1901.67 ± 47.12	4.82 ± 2.44	0.22 ± 0.07	0.76 ± 0.29	0.86 ± 0.31
	Entire Lamina	1560.69 ± 19.81 **	0.54 ± 0.11	0.10 ± 0.01	0.21 ± 0.02	0.34 ± 0.02
Fruits	Pinnatifid	1120.93 ± 20.25	0.07 ± 0.02	0.07 ± 0.00	0.14 ± 0.02	0.14 ± 0.01
	Entire Lamina	1044.08 ± 11.80 *	0.05 ± 0.00	0.06 ± 0.00	0.10 ± 0.01	0.09 ± 0.01 *

		**Pb**	**Mn**	**Mo**	**Se**	**Hg**
Stems	Pinnatifid	0.06 ± 0.01	0.63 ± 0.04	0.02 ± 0.02	0.06 ± 0.01	0.05 ± 0.00
	Entire Lamina	0.07 ± 0.00	0.57 ± 0.01	0.01 ± 0.00	0.02 ± 0.01	0.04 ± 0.00
Leaves	Pinnatifid	0.10 ± 0.01	1.14 ± 0.58	0.00 ± 0.00	0.06 ± 0.01	0.03 ± 0.00
	Entire Lamina	0.11 ± 0.03	0.13 ± 0.02	0.01 ± 0.00 *	0.02 ± 0.00 *	0.03 ± 0.00
Fruits	Pinnatifid	0.07 ± 0.03	0.03 ± 0.00	0.02 ± 0.00	0.02 ± 0.02	0.04 ± 0.00
	Entire Lamina	0.10 ± 0.01	0.02 ± 0.00	0.01 ± 0.00	0.03 ± 0.02	0.02 ± 0.00 *
